# Effect of Replacing Sugar with Non-Caloric Sweeteners in Beverages on the Reward Value after Repeated Exposure

**DOI:** 10.1371/journal.pone.0081924

**Published:** 2013-11-28

**Authors:** Sanne Griffioen-Roose, Paul A. M. Smeets, Pascalle L. G. Weijzen, Inge van Rijn, Iris van den Bosch, Cees de Graaf

**Affiliations:** 1 Division of Human Nutrition, Wageningen University, Wageningen, The Netherlands; 2 Image Sciences Institute, University Medical Center Utrecht, Utrecht, The Netherlands; 3 Research Department Sensory & Consumer Science, FrieslandCampina, Amersfoort, The Netherlands; CNR, Italy

## Abstract

**Background:**

The reward value of food is partly dependent on learned associations. It is not yet known whether replacing sugar with non-caloric sweeteners in food is affecting long-term acceptance.

**Objective:**

To determine the effect of replacing sugar with non-caloric sweeteners in a nutrient-empty drink (soft drink) versus nutrient-rich drink (yoghurt drink) on reward value after repeated exposure.

**Design:**

We used a randomized crossover design whereby forty subjects (15 men, 25 women) with a mean±SD age of 21±2 y and BMI of 21.5±1.7 kg/m^2^ consumed a fixed portion of a non-caloric sweetened (NS) and sugar sweetened (SS) versions of either a soft drink or a yoghurt drink (counterbalanced) for breakfast which were distinguishable by means of colored labels. Each version of a drink was offered 10 times in semi-random order. Before and after conditioning the reward value of the drinks was assessed using behavioral tasks on wanting, liking, and expected satiety. In a subgroup (n=18) fMRI was performed to assess brain reward responses to the drinks.

**Results:**

Outcomes of both the behavioral tasks and fMRI showed that conditioning did not affect the reward value of the NS and SS versions of the drinks significantly. Overall, subjects preferred the yoghurt drinks to the soft drinks and the ss drinks to the NS drinks. In addition, they expected the yoghurt drinks to be more satiating, they reduced hunger more, and delayed the first eating episode more. Conditioning did not influence these effects.

**Conclusion:**

Our study showed that repeated consumption of a non-caloric sweetened beverage, instead of a sugar sweetened version, appears not to result in changes in the reward value. It cannot be ruled out that learned associations between sensory attributes and food satiating capacity which developed preceding the conditioning period, during lifetime, affected the reward value of the drinks.

## Introduction

The prevalence of obesity has risen dramatically in the last decades resulting in increasing numbers of public health problems [1]. Obesity is the result of a positive energy balance i.e. energy intake exceeding the energy expenditure [2]. In recent years, governmental and public health organizations have therefore actively promoted dietary recommendations, such as increased consumption of fruit and vegetables and reduced intake of sugar [3,4]. This has led to an increase of products on the market where sugar is replaced by non-caloric substitutes, so-called ‘light’ products [5,6]. 

The reward value of a food product is partly dependent on a learned association; through repeated consumption of foods during our lifetime we learn to associate the sensory attributes of food (e.g. taste), with their physiological effect (e.g. amount of energy) and thereby learn to estimate their metabolic effects [7,8]. This gives rise to the possibility that repeatedly consuming products where the rewarding component, i.e. sugar, is withdrawn, might lead to a decreased preference for these foods compared to their higher-energy counterparts. In recent studies, it has been shown that caloric and non-caloric versions of a soft drink differentially affect taste activation in brain areas during tasting which are implicated in food intake regulation, like the amygdala and striatum [9-11]. The human brain appears to be able to differentiate between carbohydrates (sugar) and sweetness [12,13]. It is not yet known whether reducing sugar in food is affecting long-term acceptance and whether this is related to other aspects of the food, i.e. whether the product contains other nutrients. 

The primary objective of this study was to determine the effect of replacing sugar with non-caloric sweeteners in a nutrient-empty drink (soft drink) versus a nutrient-rich drink (yoghurt drink) on reward value after repeated exposure. Reward value was assessed with behavioral tasks and functional Magnetic Resonance Imaging (fMRI) measurements. It was hypothesized that the reward value of foods that are nutrient-rich would be less affected by replacing the sugar content than the reward value of foods that are nutrient-empty. Therefore, we expected that the reward value of a yoghurt drink sweetened with a non-caloric sweetener would not change after repeated exposure and would remain similar to that of the sugar sweetened version. Conversely, we hypothesized that a soft drink sweetened with a non-caloric sweetener would decrease in reward value after repeated exposure compared to its sugar-sweetened counterpart.

## Methods

### Ethics statement

This study was conducted according to the guidelines laid down in the Declaration of Helsinki and all procedures involving human subjects were approved by the Medical Ethical Committee of Wageningen University. All subjects signed an informed-consent form before participation. This study has been registered with the Dutch Trial register (NTR: 3289) at: http://www.trialregister.nl/trialreg/admin/rctsearch.asp?Term=3289.

### Subjects

Forty subjects (15 men, 25 women) with a mean±SD age of 21±2 y and BMI of 21.5±1.7 kg/m^2^ completed the study, which ran from March till June 2012. All subjects participated in the behavioral part of the study, and 18 subjects (15 men, three women) participated in the fMRI part of the study. Of the 41 subjects enrolled in the study, one subject dropped out during the first conditioning week. A supplemental flow diagram of the progress through the phases of the study is available online (Figure **S1**). We recruited healthy, normal-weight subjects, aged 18-35 y. Exclusion criteria were as follows: restrained eating (Dutch Eating Behavior Questionnaire (DEBQ), men: score >2.25, women: score >2.80 [14], lack of appetite, an energy restricted diet during the past two months, change in body weight > five kg during the past two months, stomach or bowel diseases, diabetes, thyroid disease or any other endocrine disorder, use of daily medication other than oral contraceptives, having difficulties with swallowing/eating, having taste or smell disorders, being allergic/intolerant for products under study, smoking, and for women, being pregnant or lactating. In addition, exclusive consumers or avoiders of ‘light versions’ of soft drinks and/or yoghurt drinks were excluded (assessed with questionnaire) as they might be more sensitive to the taste of specific sweeteners. In addition, for the subjects that also participated in the fMRI part of the study, exclusion criteria were contra-indications for MRI scanning. 

Potential subjects filled out an inclusion questionnaire including a medical history questionnaire. They attended a screening and practice session which included measurement of weight and height and explanation/practice of the different procedures. Subjects were unaware of the exact aim of the study; they were not informed that the drinks contained either sugar or artificial sweeteners. They were informed we were investigating the effect of repeated consumption of different beverages on satiety and were naïve to the fact that we specifically investigated reward. 

### Design

**Figure 1:**
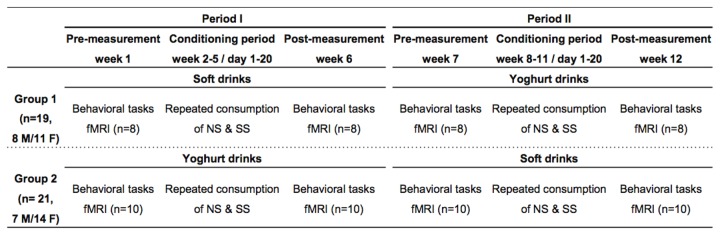
Schematic overview of study design. The study consisted of two periods, and each period had three parts: a pre-measurement, a conditioning period, and a post-measurement. In the conditioning period, subjects were offered a non-caloric sweetened (NS) and sugar sweetened (SS) versions of either a soft drink or a yoghurt drink. In period 1, subjects received either the NS and the SS versions of the soft drinks or the NS and SS versions of the yoghurt drinks. In period 2 these conditions were counterbalanced. The pre- and post-measurements assessed reward value of the drinks using behavioral tasks and fMRI. A randomized crossover design was used. The NS and SS versions of a drink were offered in semi-random order, i.e. each product was offered not more than two days in a row.

We used a randomized crossover design consisting of two periods and each period consisted of three parts: a pre-measurement, a conditioning period, and a post-measurement (Figure **1**). In the conditioning period, subjects were offered a non-caloric sweetened (NS) and sugar sweetened (SS) versions of either a soft drink or a yoghurt drink for breakfast. To enable subjects to differentiate between the drinks they were marked with a colored label (see ‘Stimuli’). In period 1, subjects received either the NS and the SS versions of the soft drinks or the NS and SS versions of the yoghurt drinks. In period 2 these conditions were counterbalanced. Subjects were randomly assigned to one of the two groups by the principal investigator, taking gender into account. The pre- and post-measurements assessed reward value (our primary outcome measure) of the drinks using behavioral tasks and fMRI. We assessed *wanting* with a choice task, (implicit) intake, and explicit question, *liking* was assessed with an explicit question and the implicit association task (IAT), and (*expected*) *satiety* was assessed with questionnaires. The brain reward responses to the NS and SS drinks were investigated with fMRI.

### Stimuli

All test foods (the NS and SS versions of the soft drink and yoghurt drink) were developed and prepared specifically for this experiment (Royal FrieslandCampina, Amersfoort, The Netherlands). The soft drinks were grape/lemon flavored and the yoghurt drinks were cherry flavored. The NS and SS versions of the drinks were closely matched in terms of appearance, odor, taste and texture. This was confirmed by sensory expert panels (separate panels for the soft and the yoghurt drinks), consisting of subjects which were screened for taste sensitivity (n=12). Characteristics of the drinks are shown in Table 1. The energy densities and sensory characteristics of the used products were all similar to products that are available in the supermarket.

**Table 1:** Characteristics of the drinks.

**Drink**		**Label**	**Energy / 100 g**	**Ingredients**
Soft drinks	NS	green or pink	0 kJ / 0 kcal	water, sucralose (0.11 g/l), fruit juice, citric acid, aroma, CO_2_
	SS	green or pink	167 kJ / 40 kcal	water, sucrose (68.6 g/l), fruit juice, citric acid, aroma and CO_2_
Yoghurt drinks	NS	yellow or blue	167 kJ / 40 kcal	skim yogurt, sucralose (0.008 g/l), acesulfame-K (0.013 g/l), fruit juice, calcium, aroma and vitamin B2, B6, B12
	SS	yellow or blue	335 kJ / 80 kcal	skim yoghurt, sucrose (6.8 g/l), fruit juice, calcium, aroma and vitamin B2, B6, B12

NS = non-caloric sweetened, SS = sugar sweetened .

All test foods (the NS and SS versions of the soft drink and yoghurt drink) were developed and prepared specifically for this experiment (Royal FrieslandCampina, Amersfoort, The Netherlands). The soft drinks were grape/lemon flavored and the yoghurt drinks were cherry flavored. The NS and SS versions of the drinks were closely matched in terms of appearance, odor, taste and texture. This was confirmed by sensory expert panels (separate panels for the soft and the yoghurt drinks), consisting of subjects which were screened for taste sensitivity (n=12).

Specific procedure for development of the soft drinks: three versions of the NS soft drinks were developed and these were tested against the SS version with a direct-comparison ranking test on sweetness and fruitiness. In addition, a sensory profiling test on other attributes was performed. The drinks were semi-monadically rated on appearance (light-dark color), odor (fruitiness, oxidation, freshness, complexity), and taste (oxidation, freshness, complexity) on a 100-mm VAS. The NS variant that was chosen did not significantly differ from the SS version in the ranking test and in the sensory profiling test: light-dark color NS 25 vs. SS 27; odor fruitiness NS 62 vs. SS 63; odor oxidation NS 32 vs. SS 32; odor freshness NS 58 vs. SS 65; odor complexity NS 60 vs. SS 63; taste oxidation NS 30 vs. SS 30; taste freshness NS 57 vs. SS 67; taste complexity NS 55 vs. SS 63.

Specific procedure for the development of the yoghurt drinks: three versions of the NS yoghurt drinks and three versions of the SS yoghurt drinks were developed and a sensory profiling test was performed. The drinks were semi-monadically rated on appearance (light-dark color), odor (sourness, fruitiness), taste (sourness, sweetness, fruitiness), mouth feel (thickness), and aftertaste (sourness, liquorice) on a 100-mm VAS to determine the best match. The match that was choses did not significantly differ on any of the attributes: light-dark color NS 48 vs. SS 53; odor sourness NS 32 vs. SS 29; odor fruitiness NS 78 vs. SS 78; taste sourness NS 30 vs. SS 30; taste sweetness NS 73 vs. SS 68; taste fruitiness NS 74 vs. SS 73; mouthfeel thickness NS 46 vs. SS 46; aftertaste sourness NS 30 vs. SS 33; aftertaste liquorice NS 3 vs. SS 0.

To enable subjects to differentiate between the drinks they were paired with a colored label. For the soft drinks, half of the subjects received the NS version with a green label the other half with a pink label (and vice versa for the SS version). For yoghurt drinks: half of the subjects received the NS version with a yellow label and the other half with a blue label (and vice versa for the SS version). This procedure of pairing drinks with a colored label has been shown to enable ‘energy learning’ [15]. During the measurements and the conditioning period subjects were offered a fixed portion of the test foods: the males received 400 mL (NS soft drink: 0 kJ / 0 kcal, SS soft drink: 669 kJ / 160 kcal, NS yoghurt drink: 669 kJ / 160 kcal, SS yoghurt drink: 1339 kJ / 320 kcal) and the females 300 mL (NS soft drink: 0 kJ / 0 kcal, SS soft drink: 502 kJ / 120 kcal, NS yoghurt drink: 502 kJ / 120 kcal, SS yoghurt drink: 1004 kJ / 240 kcal). 

### Procedure

#### Conditioning period

The conditioning period lasted 4 weeks (=20 exposures). Subjects were offered a NS or a SS version of either the soft drink or the yoghurt drink. Each version of a drink was offered 10 times in semi-random order, i.e. each product was offered not more than two days in a row. During the conditioning period subjects came to the research center at the Wageningen University (Wageningen, The Netherlands) on weekdays between 7:30 and 9:00 a.m. in a fasted state (no eating or drinking anything except water after overnight fast). Upon arrival subjects filled out an appetite questionnaire, consisting of 5 dimensions: hunger, fullness, prospective consumption, desire to eat, and thirst. The 9-point scale was anchored with ‘not at all’ to ‘extremely’. Next, subjects consumed their drink with a straw. After finishing they rated their appetite and were then free to leave. During the day subjects reported at what time they had their first eating episode and what they consumed. Subjects were instructed not to eat for at least 1 hr after consumption of the drinks.

#### Pre- and post-measurements - Behavioral tasks

All subjects were tested between 7:00 and 8:30 in fasted state in an isolated sensory booth. When subjects arrived at the research center at the Wageningen University (Wageningen, The Netherlands) they were given specific instructions shown on a computer screen. All test sessions started with subjects filling out an appetite questionnaire, consisting of a hunger and thirst question. The 100-unit visual analogue scale (VAS) was anchored with ‘not at all’ to ‘extremely’. 

Next, subjects were offered the two drinks (similar cups and amounts as in the condition period): either the NS and the SS versions of the soft drinks or the NS and the SS versions of the yoghurt drinks (depending on the ‘period’). Subjects were instructed to taste the drinks and rate their ‘explicit liking’ (“How pleasant do you find the taste of this drink right now?”), their ‘explicit wanting’ (“How much do you want to consume this drink right now?”), and their ‘expected satiety’ (“How filling do you think this drink is?”). These three questions per drink were accompanied with a clear identifiable picture of the drink and were asked in randomized order. This was followed by a computerized ‘choice task’. Subjects were shown a paired presentation of two drinks where they had to select their most wanted drink (“select the drink which you would most want to drink right now”). The first four presentation pairs consisted of pictures of red bull energy drink, diet coke, milkshake and orange juice, presented in random order (e.g. subjects had to choose between red bull energy drink vs. diet coke, or between diet coke vs. orange juice). During the fifth presentation, pictures of the NS and SS versions of the drinks, with the colored labels clearly visible, were shown and subjects had to choose the drink which they would most want to drink right now. During the choice task the chosen drink and the reaction time with which this drink was chosen (implicit wanting) were measured. During the whole procedure subjects were allowed to consume as little or as much of the drinks as they wanted. After the choice task procedure the two drinks were collected and intake (g) was measured. Subjects were not aware of the fact that intake was measured (implicit intake). 

After the drinks were collected subjects proceeded with an implicit association task (IAT) [16]. The IAT is a refined tool to measure implicit associations between concepts that are related to attitudes and behavior; i.e. it provides insights into subconscious liking. In the IAT subjects responded to a series of items that were classified into four categories, the NS category, the SS category, a positive category and a negative category.

In the right-hand and left-hand corner of the screen the NS and SS categories were coupled with the positive and negative categories. Subjects were presented with a series of stimuli in the center of the screen consisting of either a picture or a word and they were asked to categorize these to the appropriate corner by pressing the appropriate key (left or right). Of both the NS and the SS versions of the drink five different pictures were used. For the positive associations the following attributes were used: joy, love, peace, happiness, freedom (in Dutch: plezier, liefde, vrede, geluk, vrijheid). For the negative associations the following attributes were used: sadness, hate, war, unhappiness, captivity (in Dutch: verdriet, haat, oorlog, ongeluk, gevangen). Subjects then performed a second task where the pairing of the categories were switched. The IAT produces measures derived from latencies of responses to these two tasks. These measures are interpreted in terms of association strengths by assuming that subjects respond more rapidly when the concept and attribute mapped onto the same response are strongly associated (e.g. a drink that is rewarded higher with the positive category) than when they are weakly associated (e.g. a drink that is rewarded higher with the negative category). After finishing the test subjects were free to leave.

#### Pre- and post-measurements - fMRI

On a separate occasion, but in the same week as the behavioral task, 18 subjects of the total group were scanned between 7:00 and 11:00 a.m at the Hospital de Gelderse Vallei (Ede, The Netherlands). All experimental measurements of one individual took place at the same time. Subjects were instructed to have refrained from eating at least 3h before the test. The scan session consisted of 3 functional runs during which 262 functional volumes were acquired using a T_2_*-weighted gradient echo images (EPI), acquired with blood-oxygen level-dependent (BOLD) contrast on a 3-Tesla Siemens Magnetom Verio MRI scanner (Siemens, Erlangen, Germany) equipped with a 32-channel head coil. Whole-brain fMRI data were obtained with a T_2_*-weighted 2D echo-planar imaging sequence (TR=2140ms, TE=25ms, 90° flip angle, FOV=192x192mm, 43 axial slices, ascending order, voxel size 3x3x3 mm). The imaging volume was tilted at an oblique angle of 30° to the anterior-posterior commissure line to reduce signal dropout in orbitofrontal and ventral temporal lobes [17]. In addition, a high-resolution T_1_-weighted anatomical MRI scan (MPRAGE, TR=1900ms, TE=2.26ms, 9° flip angle, FOV= 256×256mm, 192 sagittal slices, voxel size=0.5×0.5×1mm) was acquired after the second functional run. Each functional run consisted of 5 taste events each for SS, NS, tomato juice and water, leading to a total of 15 taste events per stimulus. During scanning, subjects alternately tasted 2 mL of the NS, the SS, tomato juice and water. They tasted it for 11 sec while a picture of the drink was shown, followed by a visual cue for swallowing (3 s) and a 4-s rinse with water. Tomato juice (Appelsientje Zontomaat, Ede, The Netherlands) was chosen because of its differing sensory characteristic than the target drinks, to reduce sensory specific satiety [18]. Water was used as a control stimulus to be able to account for general taste and swallow effects. During every functional run liking and wanting of all stimuli was rated on a 9-point hedonic scale with the use of a button box (6 s), leading to a total of 3 liking and 3 wanting ratings per stimulus. 

### Analysis

Data are presented as mean values with standard errors unless otherwise specified.

#### Behavioral tasks

For the choice task chi-squared analyses were performed (NS vs. SS). All the other behavioral measures (implicit wanting, implicit intake, explicit wanting, explicit liking, results of the IAT, and expected satiety) were analyzed by means of ANOVA (mixed model procedure) with drink type (soft and yoghurt), sweetener type (NS and SS) and time (before and after conditioning) as independent variables. For both the implicit wanting and the results of the IAT, the analyses included all trials with latency longer than 300 ms and shorter than 4,000 ms. We log-transformed response latencies prior to aggregating data (untransformed latency means are reported in text). The results obtained during the conditioning period (hunger ratings and time to first consumption) were analyzed by means of ANOVA (mixed model procedure) with drink type (soft and yoghurt) and sweetener type (NS and SS) as independent variables. In all analyses, both main effects and interactions between the independent variables were analyzed. In addition, subjects were included in all models as random factor. Tukey’s test was used for post-hoc comparisons. Analyses were conducted with the use of SAS, 9.1 (SAS Institue, Inc., Cary, NC, USA). 

#### fMRI

fMRI data were preprocessed and analyzed with the SPM8 software package (Wellcome Department of Imaging Neuroscience, London, UK) in conjunction with the MarsBar toolbox (http://marsbar.sourceforge.net/) run with MATLAB 7.12 (The Mathworks Inc, Natick, MA). The functional volumes of every subject were realigned, globally normalized to Montreal Neurological Institute space (MNI space), and spatially smoothed with an isotropic Gaussian kernel of 8 mm full width at half maximum. Seven conditions were modeled: delivery of NS, SS, tomato juice, water, swallowing, rinsing and rating. The responses to swallowing, rinsing and rating were neglected in further analyses. To regress out motion-related variance, the motion-correction parameters from the realignment procedure were added to the model as regressors.

For every subject, parameters were estimated for four comparisons per drink type (referred to as contrasts), contrast images were calculated for tasting NS or SS versus the control condition (water), before and after conditioning. Due to measurement errors the soft drink data of two subjects were disregarded because of insufficient data quality. 

To test our hypothesis a whole-brain statistical F-map was created by performing an ANOVA with sweetener type (NS and SS) and time (before and after conditioning) as independent variables per drink type (Table **S1** and Table **S2**). We used a region of interest (ROI) approach that combined a priori anatomical areas of interest with a functional criterion based on a minimum level of responsiveness to food cues and learning [19,20]. On the basis of previous research [9,10] anatomic areas of interest included the orbitofrontal cortex (OFC), amygdala, thalamus, striatum (pallidum, putamen, caudate), cingulate gyrus, hippocampus, precentral gyrys and taste areas identified in a meta-analysis (we used the complete activation map available at http://flavor.monell.org/~jlundstrom/index_ALE.html) [21]. Mask images were obtained from the WFU Pickatlas 9 [22]. With the exception of the cingulate and precentral gyrus, all masks images were dilated one voxel to account for anatomical variation and smoothing effects. To identify functional ROIs (fROIs) both created maps were thresholded at a significance level of p<0.05 and a cluster size k>8 contiguous voxels. The identified fROIs for tasting soft drinks were: OFC, thalamus, caudate, middle cingulum, right precuneus, hippocampus, precentral gyrus, insula, and rolandic operculum. The identified fROIs for tasting yoghurt drinks were: OFC, amygdala, thalamus, caudate, putamen, middle cingulum, hippocampus, precentral gyrus, and rolandic operculum. The mean beta value in each fROI was calculated with the use of MarsBar, and submitted to an ANOVA (mixed model procedure) in SAS. This allowed us to test the effects of sweetener type (NS and SS) and time (before and after conditioning) within each fROI for each drink type. This technique represents an unbiased approach to test a priori hypotheses and avoids problems of circularity [19,20]. 

## Results

### Behavioral tasks

#### Wanting

**Figure 2:**
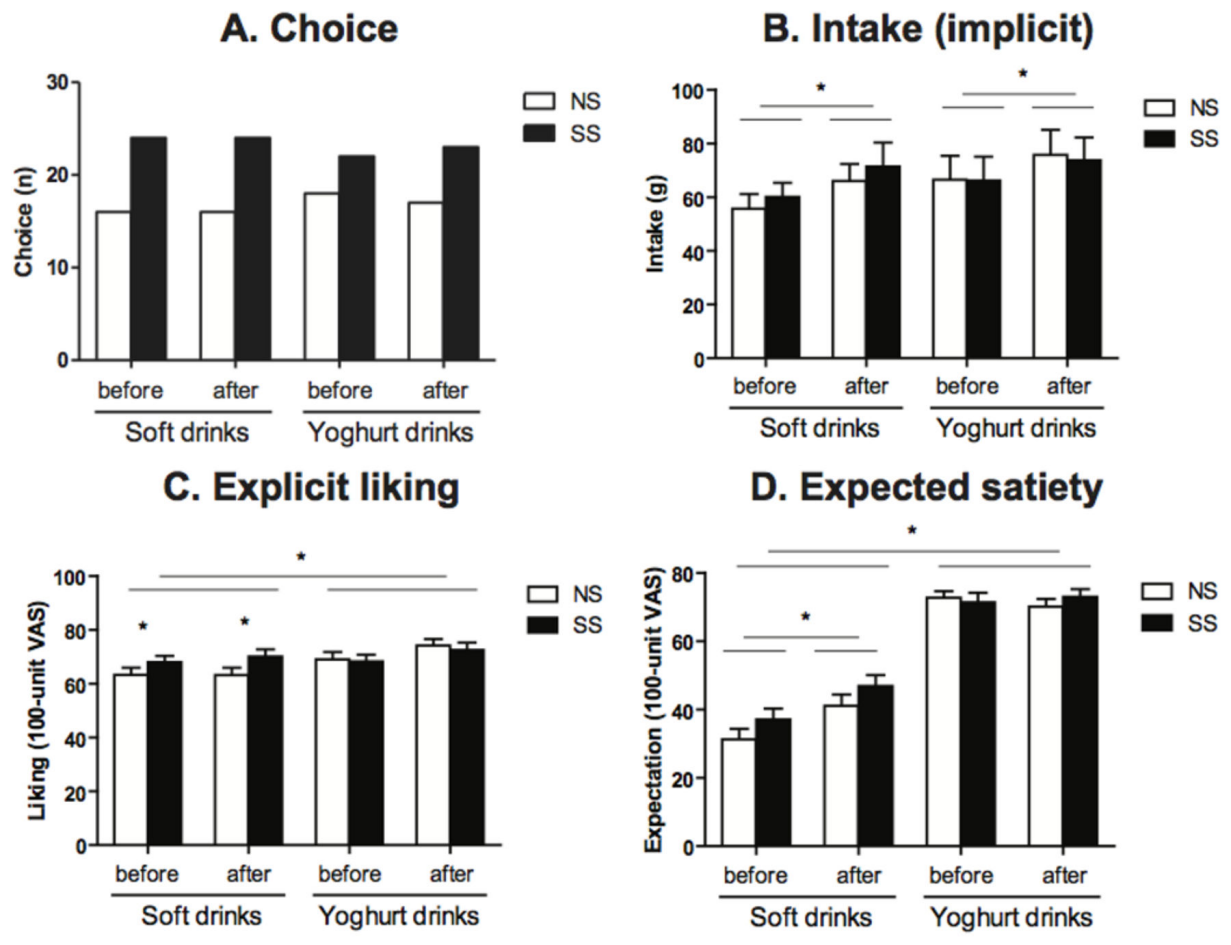
Results of the behavioral tasks for the NS (◻) and SS (◼) soft drinks and yoghurt dinks. (A) Choice: there was a main significant effect for sweetener type (p<0.05): the SS drinks were chosen more often than the NS drinks. (B) Total (implicit) intake (g): there was a main significant effect of time (p<0.05): intake was greater after the conditioning period than before. (C) Explicit liking: there was a main significant effect of drink type (p<0.01): the yoghurt drinks were more explicitly liked than the soft drinks. There was a significant interaction between drink type and sweetener type: the SS soft drink was more liked than the NS soft drink (p<0.05). (D) Expected satiety: there was a significant main effect of drink type (p<0.0001): the yoghurts drinks were expected to be more satiating than the soft drinks. There was a significant interaction between drink type and time (p<0.01): after the conditioning period the soft drinks were rated as more satiating. Values are means ± SEMs (n=40). For the choice tasks chi-squared analyses were performed. All others were analyzed by means of ANOVA (mixed model procedure).

Overall the SS drinks were chosen more often than the NS drinks in the choice task [c^2^(1, n=160)=4.23, p<0.05] (Figure **2A**). When analyzed separately per drink type, there was a trend that the SS soft drink was chosen more often than the NS soft drink [c^2^(1, n=80)=3.20, p=0.07]. This was not shown for the yoghurt drinks. There was no effect of time on choice. The speed with which the choice was made (implicit wanting) did not differ between drink types or sweetener types. Subjects responded faster after the conditioning period compared with before [F(1,39)=12.58, p<0.01]. There were no significant interactions. 

Subjects tended to (implicitly) consume more of the yoghurt drink than of the soft drink [F (1,39)=3.26, p=0.08] (Figure **2B**). The intake of NS drinks and SS drinks did not differ. Consumption was greater after the conditioning period than before [F(1,39)=5.77, p<0.05]. There were no significant interactions. 

The explicit wanting ratings showed that subjects wanted the yoghurt drinks more than the soft drinks [F(1,39)=22.14, p<0.0001]. There was no effect of sweetener type or time on explicit wanting. There were no significant interactions.

#### Liking

Subjects explicitly liked the yoghurt drinks more than the soft drinks [F(1,39)=9.14, p<0.01] (Figure **2C**). There was no main effect of sweetener type on explicit liking. However, there was a significant interaction between drink type and sweetener type [F(1,39)=4.77, p<0.05], with post hoc (Tukey’s) analyses showing that the SS soft drink was more liked than the NS soft drink. Subjects tended to like the drinks more after the conditioning period than before [F(1,39)=3.17, p=0.08]. 

In the IAT, the speed of associating the drinks with positive and negative attributes did not differ between drink types and sweetener types. Subjects responded faster after the conditioning period than before [F(1,39)=5.95, p<0.05] (Table 2). There were no significant interactions.

**Table 2:** Results of the IAT – Mean (SD) latency for each condition × category (positive and negative).

**Drink**			**Positive (ms)**	**Negative (ms)**
Soft drinks	Pre-measurement	NS	652 (151)	694 (184)
		SS	692 (197)	691 (221)
	Post- measurement	NS	636 (163)	692 (228)
		SS	637 (134)	691 (226)
Yoghurt drinks	Pre-measurement	NS	673 (171)	681 (196)
		SS	695 (213)	674 (133)
	Post- measurement	NS	648 (143)	629 (125)
		SS	657 (183)	644 (136)

NS = non-caloric sweetened, SS = sugar sweetenedNS: non-caloric sweetened; SS: sugar sweetened

#### Expected satiety

Subjects expected the yoghurts drinks to be more satiating than the soft drinks [F(1,39)=323.7, p<0.0001] (Figure **2D**). The SS drinks tended to have a higher expected satiety than NS drinks [F(1,39)=3.2, p=0.08). There was a significant interaction between drink type and time [F(1,39)=8.0, p<0.01), with post hoc (Tukey’s) analyses showing that after the conditioning period, the soft drinks were rated as more satiating than before. 

#### Satiety scores in the conditioning period

**Figure 3:**
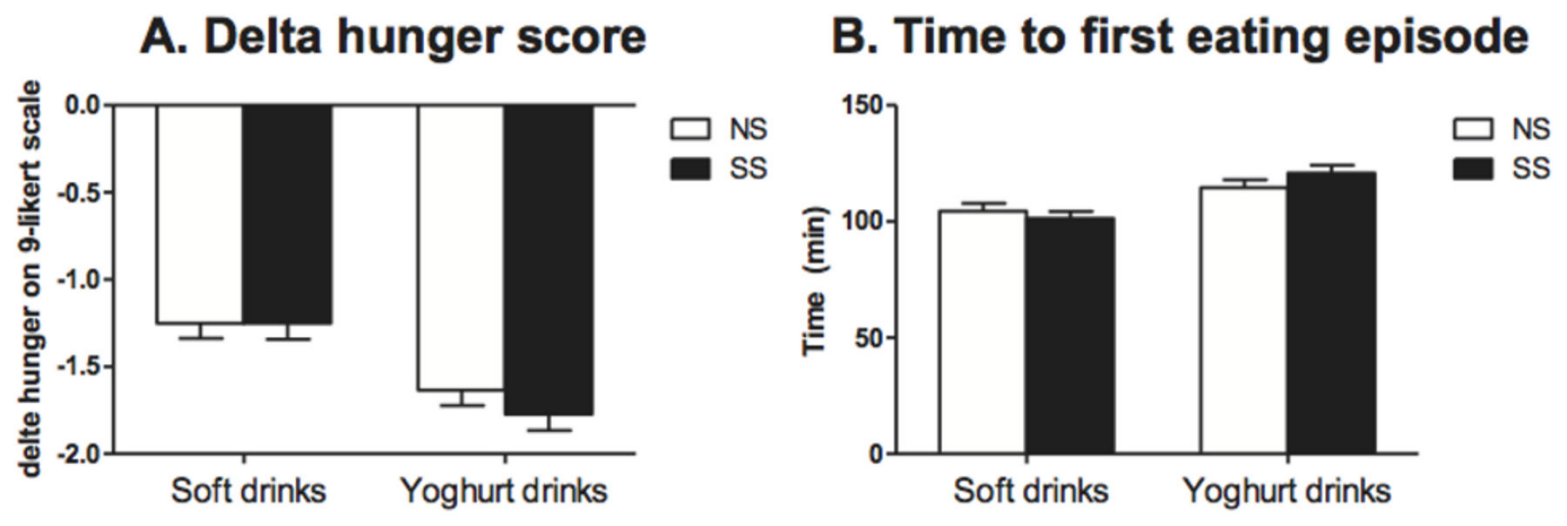
Satiety scores in the conditioning period (A) The average delta scores of the hunger ratings in the conditioning period (hunger rating after consumption minus hunger rating before consumption) for the NS (◻) and SS (◼) soft drinks and the yoghurt drinks: there was a significant main effect of drink type (p<0.0001): the yoghurt drinks reduced hunger more than the soft drinks. (B) The average time until subjects ate their first item after consumption in the conditioning period for NS (◻) and SS (◼) soft drinks and the yoghurt drinks: there was a significant main effect of drink type (p<0.0001): after the yoghurt drinks the average time was longer than after the soft drinks. Values are means ± SEMs (n=40). Analyses were performed by means of ANOVA (mixed model procedure).

Analyses on the average delta scores of the hunger ratings in the conditioning period (hunger rating after consumption minus hunger rating before consumption) showed that the yoghurt drinks reduced hunger more than the soft drinks [F(1,39)=52.45, p<0.0001]. There was no effect of sweetener type, and there were no significant interactions (Figure **3A**). 

The average time until subjects ate their first item after consumption of the drinks in the conditioning period was longer after the yoghurt drinks than after the soft drinks [F(1,32)=23.22, p<0.0001]. Again, there was no effect of sweetener type or interactions (Figure **3B**).

### fMRI

**Figure 4:**
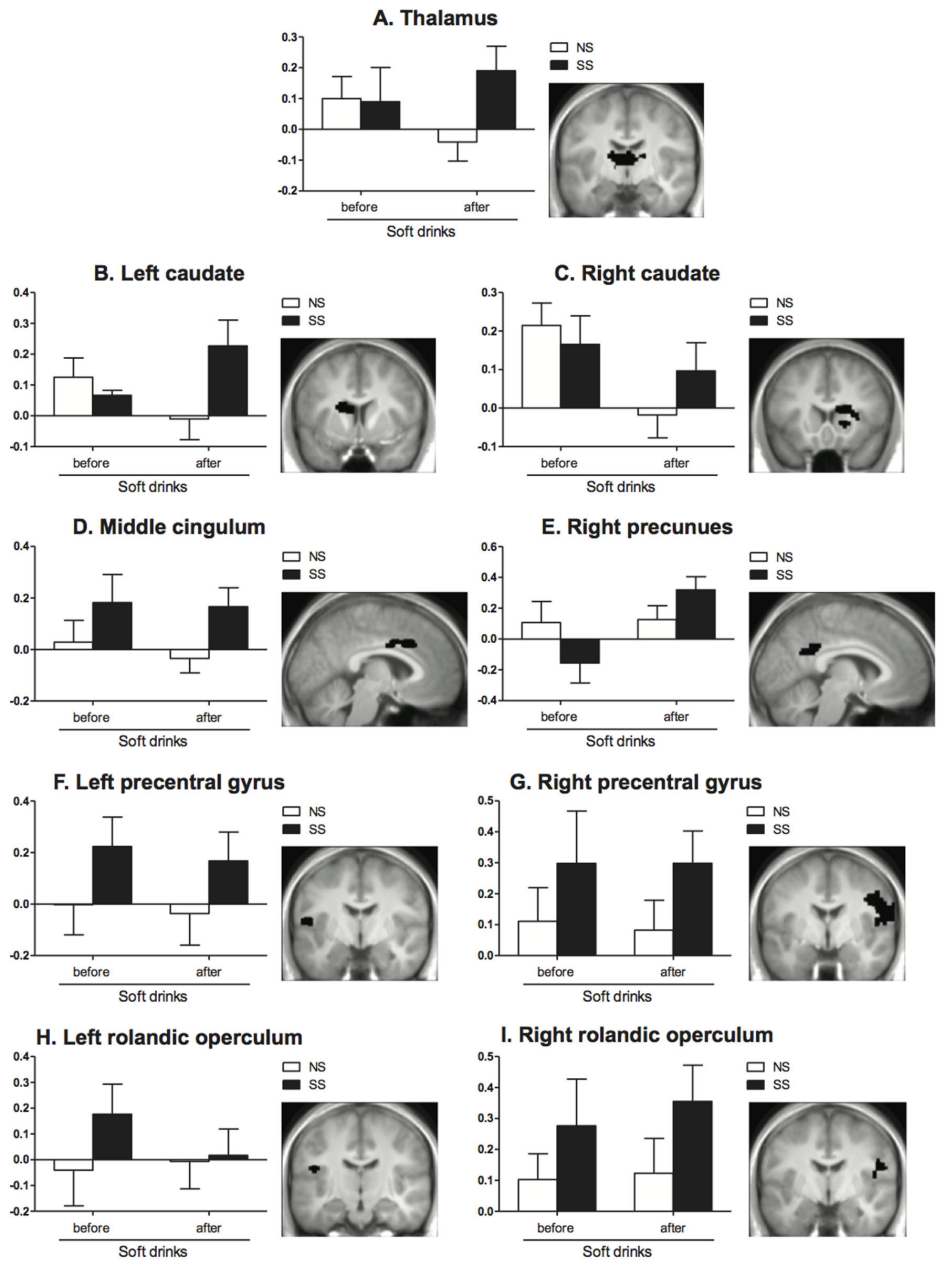
Mean beta value for each fROI after tasting the NS (◻) and SS (◼) soft drinks before and after conditioning for (A) the thalamus: the main effect of sweetener type was a non-significant trend (p=0.05). (B) the left caudate: there was a significant interaction between sweetener type and time (p<0.05): however post-hoc analyses showed that the difference between the NS and SS version after conditioning was a non-significant trend (p=0.09). (C) the right caudate: no significant differences (D) the middle cingulum: there was a significant main effect of sweetener type (p<0.05). (E) right precuneus: there was a significant interaction between sweetener type and time (p<0.05): post-hoc analyses showed that taste activation was increased for the SS soft drink after conditioning (p<0.01). (F) left precentral gyrus: there was a significant main effect of sweetener type (p<0.05). (G) right precentral gyrus: there was a significant main effect of sweetener type (p<0.05). (H) left rolandic operculum: no significant differences (I) right rolandic operculum: there was a significant main effect of sweetener type (p<0.05). Values are means ± SEM (n=16). Analyses were performed by means of ANOVA (mixed model procedure). Next to each graph the corresponding fROI is shown in black on a representative slice of the mean anatomical MRI of all fMRI subjects.

Soft drinks: The fROI analyses of the brain response to tasting showed that the areas that differentially responded to NS and SS versions of the soft drink were the middle cingulum, precentral gyrus, rolandic operculum, and thalamus, although in the latter case the effect was a non-significant trend (Table 3 and Figure **4**). 

**Table 3:** Identified fROIs and results of analysis on mean beta value in each fROI for tasting soft drinks.^a^

**fROI**	**Peak voxel coordinates of fROI**			**BA**	**Cluster size (voxels)**	**F peak voxel**	**Main effect sweetener type**	**Sweetener type x Time**
	**x**	**y**	**z**				*p*	*p*
*OFC*								
L Medial Frontal gyrus	-6	59	-14	11	72	5.4	0.91	0.34
L Inferior Frontal gyrus	-39	32	-5	47	9	2.8	0.95	0.18
R Middle Frontal gyrus	27	62	-8	11	11	3.9	0.16	0.49
R Superior Frontal gyrus	24	35	-17	11	10	3.4	0.93	0.46
R Medial Frontal gyrus	9	59	-14	11	9	3.0	0.53	0.11
R Superior Temporal Pole	51	17	-2	38	14	3.8	0.80	0.13
*Thalamus*								
Thalamus	-6	-10	10	-	336	8.1	0.07	0.05
*Striatum*								
L Caudate	-18	8	16	-	136	6.6	0.20	**0.04**
R Caudate	18	17	13	-	255	8.1	0.50	0.11
*Cingulate gyrus*								
Middle Cingulum	9	-1	34	24	240	8.9	**0.02**	0.73
R Precunues	9	-52	25	23	105	6.8	0.68	**0.01**
*Hippocampus*								
L Hippocampus	-33	-34	-5	37	27	6.6	0.70	0.08
R Hippocampus	30	-37	4	37	9	4.7	0.29	0.86
*Precentral Gyrus*								
L Precentral gyrus	-51	-10	7	48	58	6.4	**0.01**	0.89
L Precentral gyrus	-51	-16	28	48	191	4.2	0.17	0.69
L Precentral gyrus	-24	-25	52	-	15	3.4	0.29	0.14
R Precentral gyrus	48	-4	34	4	326	6.4	**0.01**	0.84
R Precentral gyrus	36	-19	46	3	51	4.7	0.21	0.12
*Taste map*								
L Insula	-42	-1	4	48	12	4.6	0.12	0.70
R Insula	33	17	10	48	55	5.7	0.23	0.62
L Rolandic operculum	-48	-16	19	48	24	4.5	0.09	0.17
R Rolandic operculum	48	-7	13	48	49	5.6	**0.01**	0.69

^a^ The F map was thresholded at F=2.53, p<0.05, uncorrected for multiple comparisons, with a cluster extent threshold k>8 contiguous voxels. BA=Brodmann areas. L = Left, R=Right hemisphere

There was a significant interaction between sweetener type and time in the left caudate [F(1,15)=5.09, p<0.05] and the right precuneus [F(1,15)=7.69, p<0.05]. Post-hoc analyses showed, however, that for the left caudate the difference between the NS and SS version after conditioning was a non-significant trend. In the right precuneus taste activation was increased for the SS soft drink after conditioning.

For the soft drinks there was no effect of sweetener type or time on the liking and wanting ratings obtained in the scanner (mean±SD liking NS before: 7.1±0.9; NS after: 6.9±0.7; SS before: 7.2±1.0; SS after: 7.1±0.9). Pearson’s correlation analyses of the association between responses in fROIs that differentially responded to NS and SS versions of the soft drink and the liking and wanting ratings obtained in the scanner showed that only taste activation in the left precentral gyrus correlated weakly with subjective ratings (liking r_64=_0.24, p=0.06); wanting r_64=_0.31, p<0.05).

Yoghurt drinks: The fROI analyses of the brain response to tasting showed that there were no areas that responded differentially to NS and SS versions of the yoghurt drink (Table **S3**). There was a significant interaction between sweetener type and time in one area within the precentral gyrus, whereby taste activation was increased for the NS yogurt drink after conditioning.

For the yoghurt drinks there was no effect of sweetener type or time on the liking and wanting ratings obtained in the scanner (mean±SD liking NS before: 7.5±0.8; NS after: 7.3±1.0; SS before: 7.5±0.9; SS after: 7.2±1.4). 

## Discussion

This study investigated the effect of replacing sugar with non-caloric sweeteners in a nutrient-empty drink (soft drink) and in a nutrient-rich drink (yoghurt drink) on reward value after repeated exposure. 

### Results of the behavioral tasks showed that conditioning did not affect the reward value of the

NS and SS versions of the drinks. Overall subjects preferred the yoghurt drinks to the soft drinks. In addition, they expected yoghurt drinks to be more satiating; they reduced hunger more, and delayed the first eating episode more. There was also an effect of sweetener type: overall the SS drinks were preferred over NS drinks in the choice task and the SS soft drinks were explicitly liked more than the NS soft drinks. But there were no differences between the SS and the NS drinks on (implicit) intake, explicit wanting, results of the IAT and expected satiety. Conditioning did not influence these effects i.e. the reward value of the drinks did not decrease in the absence of energy from sugar after repeated exposure. These results did not concur with our hypothesis as we had expected that the reward value of foods that are nutrient-rich (yoghurt drinks) would be less affected by replacing the sugar content than the reward value of foods that are nutrient-empty (soft drinks).

Many studies have shown that people are able to learn about the satiating capacity of a food after repeated consumption (e.g. 23,24). However, a recent review by Yeomans showed that studies investigating this phenomenon show mixed results [25]. In our study we chose to use familiar products with familiar tastes; our products all have similar counterparts that are commercially available. By using familiar stimuli we were able to investigate the process of ‘unlearning’ – will repeated exposure to a product, with reduced energy content, change the previously learned association between the sensory properties of the food and its (expected) satiation and reward value? Our results show that this did not occur; i.e. the association between the sensory properties of the drinks and their reward value was not unlearnt in the absence of energy. 

Our results do show that from the start of the experiment, the - thicker - yoghurt drinks were expected to be more satiating; they reduced hunger more, and delayed the first eating episode more than the - thinner - soft drinks, despite similar energy content of the NS yoghurt drink and the SS soft drink. This suggests that the sensory aspects of the drink, like for instance its viscosity, influenced (expected) satiety and reward value more than the energy content, and that these sensory effects were robust over repeated exposure. This is in accord with studies which show that texture has a large effect on satiety expectations (e.g. 26,27). In addition, the association between dairy and energy is already learned during infancy which is the most sensitive period for learning associations between sensory signals and metabolic consequences [28]. Our result suggests that learned associations between the sensory attributes of a food and its satiating capacity are quite robust and not easily adapted if energy content is altered. Aside from the enhanced satiety effect, the yoghurt drink had in general also a higher reward value than the soft drinks (higher wanting and liking). This could be due to the fact that we conducted our sessions in the morning and yoghurt drinks were considered a more suitable replacement for breakfast.

As mentioned earlier, we chose to use familiar products in familiar volumes to enhance the ecological validity of our results. The difference in energy between the NS and the SS version for the males was 670 kJ/160 kcal and for the females 502 kJ/120 kcal, which might be viewed as a small difference. The recent review of Yeomans [25] however shows that these quantities have been used in earlier studies where conditioning results have been reported. In addition, instead of using the more traditional approach of using different flavors to enable ‘unlearning’, we chose to use colored labels as a discriminator as previous studies have shown food likes and preferences can be developed using this approach [15]. Our main reason for choosing to use NS and SS variants with the same flavor was to miminize the chance that the initial reward value would be different. 

It is however interesting that, independent of conditioning, the SS soft drinks were explicitly liked more and tended to be chosen over the NS drink in the choice task. In a study by Zandstra et al. [15] it was shown that after five exposures to a high- or low-energy drink, subjects chose the high-energy drink significantly more often than the low-energy drink which made the authors suggest that they found a conditioned preference for a (energy) reward. Unfortunately, this study did not include a baseline measurement, which in light of our findings raises the question whether there was a learning effect or whether there was already a preference before conditioning. 

Our results show that subjects already preferred the SS soft drink over the NS soft drink at the pre-measurement although the NS and SS versions of the drinks were carefully tested by sensory expert panels prior to the study to ensure a similar profile. As we chose to perform sensory analysis of the drinks by expert panels rather than by the subjects in the main experiment, to prevent that subjects would focus their attention on the sensory aspects of the drinks, we cannot exclude the possibility that individual differences in taste sensitivity for e.g. sweeteners account for the preference for the sugar-sweetened drinks. In our study we used several behavioral measures to assess food reward. The results show that the SS drinks were preferred over NS drinks in the choice task and the SS soft drinks were explicitly liked more than the NS soft drinks. But there were no differences between the SS and the NS drinks on (implicit) intake, explicit wanting, results of the IAT and expected satiety. It has been shown in earlier studies that rank order testing appears to be more sensitive in discriminating between products than nomadic ratings [15,29]. In addition, we added fMRI measurements which gave us the opportunity to further investigate the mechanisms underlying changes in the behavioral measures. The fMRI data showed that the only areas that instantly differentially responded to SS and NS drinks, were the middle cingulum, the precentral gyrus and rolandic operculum, and only in soft drinks. There were some indications that conditioning with the soft drinks affected taste activation in the striatum, thalamus and right precuneus (energy x time interaction). However, these effects were mostly trends (left caudate and thalamus) and non-significant (right caudate) and therefore need to be interpreted with caution. For the yoghurt drinks we did not see a difference in responses in any brain area. Several fMRI studies have shown that the brain can differentiate between sugar and sweeteners in several reward areas [9,10]. It has been suggested, however, that these effects might be modulated by the frequency of artificial sweetener use [11,30]. We excluded extreme diet product users which could have interfered with our results. In addition, the reported differences between sugar and sweeteners on taste activation are not consistent. It appears that the outcomes of these kind of studies depend heavily on between-study differences in experimental design such as the type, number, and hunger state of subjects, the type of stimuli and the type of administration [9,11,30]. Although this makes it challenging to draw clear conclusions and warrants careful interpretations, such studies do advance our understanding of the complexity of the neural mechanisms underlying the regulation of eating behavior. 

Within our study it was interesting that the majority of areas that differently responded to the SS and NS versions of the soft drinks (the precentral gyrus and rolandic operculum) were in the primary sensory areas. This leads us to assume that indeed, although the drinks were matched on sensory characteristics, the taste differences associated with the use of non-caloric sweeteners were not completely covered. This may have caused the preference for the SS drinks, presumably due to the subjects' prior experience with SS and NS drinks and the flavor differences between sucrose and sucralose. That this effect is larger in the soft drinks concurs with the brain data. Future studies should include prior taste measurements among study participants to take individual differences in taste sensitivity into account. 

It is still interesting that although it appeared there were taste differences, enhancing the discrimination factor between the NS and the SS versions of the drinks, this was not translated into ‘unlearning behavior’. i.e., conditioning with the SS and the NS version had similar impact on feelings hunger after drink consumption and on the average time to eating the first item. There is currently still a lot of discussion regarding the role of low-caloric sweeteners in weight management [31-41].. The finding that learned satiety and food preference is not completely dependent on energy content suggests that the use of products with low-caloric sweeteners might be effective for weight management. In line with this, many studies have concluded that long-term, high-quality, adequately powered randomized controlled trials are required to assess the relationship between the use of non-caloric sweeteners and weight control (e.g. 33,40). 

To conclude, our study showed that repeated consumption of a non-caloric sweetened beverage, instead of a sugar sweetened version, appears not to result in changes in the reward value. It cannot be ruled out that learned associations between sensory attributes and food satiating capacity which developed preceding the conditioning period, during lifetime, affected the reward value of the drinks.

Our data indicate that the learned associations between sensory attributes and food satiating capacity are quite robust and difficult to alter. These results need to be confirmed in future experiments.

## Supporting Information

Figure S1 **Flow diagram of the progress through the phases of the study.**
(TIFF)

Table S1 **Whole-brain statistical F-map with sweetener type and time as independent variables for tasting soft drinks.**
(DOCX)

Table S2 **Whole-brain statistical F-map with sweetener type and time as independent variables for tasting yoghurt drinks.**
(DOCX)

Table S3 **Identified fROIs and results of analysis on mean beta value in each fROI.** For tasting yoghurt drinks.
(DOCX)
