# Supplementary figures and images for: Effect of Replacing Sugar with Non-Caloric Sweeteners in Beverages on the Reward Value after Repeated Exposure
Source: PLoS One. 2013 Nov 28;8(11):e81924. doi: 10.1371/journal.pone.0081924 (PMC3842969; doi:10.1371/journal.pone.0081924)

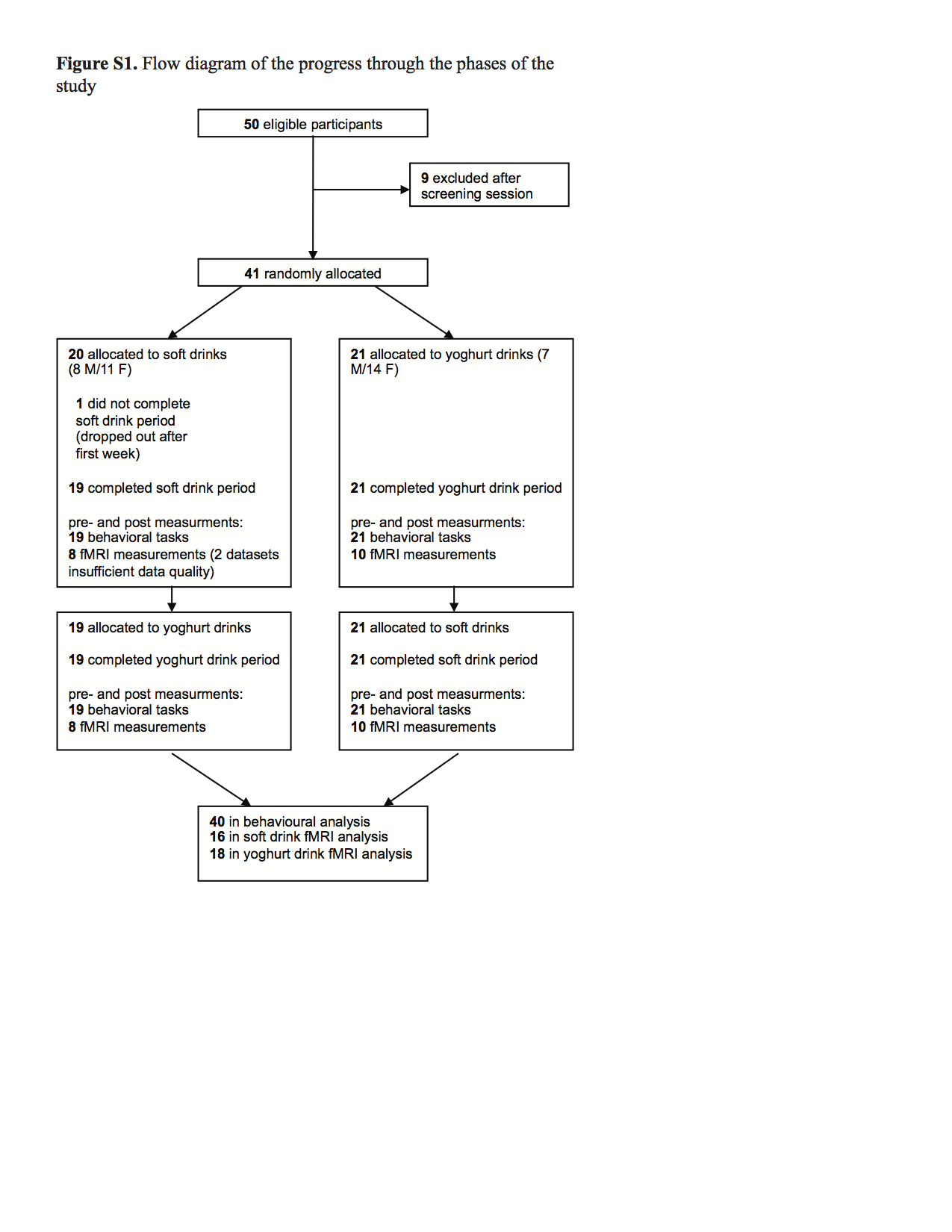

Supplement: Figure S1 — Flow diagram of the progress through the phases of the study. (TIFF) [file pone.0081924.s001.tiff]
