# Supplementary material for: Effect of Replacing Sugar with Non-Caloric Sweeteners in Beverages on the Reward Value after Repeated Exposure
Source: PLoS One. 2013 Nov 28;8(11):e81924. doi: 10.1371/journal.pone.0081924 (PMC3842969; doi:10.1371/journal.pone.0081924)
Supplement: Table S1 — Whole-brain statistical F-map with sweetener type and time as independent variables for tasting soft drinks. (DOCX) [file pone.0081924.s002.docx]

**Table S1.** Whole-brain statistical F-map with sweetener type and time as independent variables for tasting soft drinks ^a^

| **Brain region** | **Peak voxel coordinates** | | | **BA** | **F-score** | **z-score** |
| --- | --- | --- | --- | --- | --- | --- |
|  | **x** | **y** | **z** |  |  |  |
| L Medial OFC | -3 | 56 | -11 | 11 | 7.3 | 3.8 |
| L Thalamus | -6 | -10 | 10 | - | 8.1 | 4.0 |
| L Caudate | -18 | 8 | 16 | - | 6.6 | 3.6 |
| R Caudate | 18 | 17 | 13 | - | 8.1 | 4.0 |
| R Middle Cingulum | 9 | -1 | 34 | 24 | 8.9 | 4.3 |
| R Precuneus | 9 | -52 | 25 | 23 | 6.8 | 3.6 |
| L Hippocampus | -33 | -34 | -5 | 37 | 6.6 | 3.6 |
| L Superior Temporal gyrus | -48 | -13 | 4 | 48 | 8.2 | 4.1 |
| Lingual gyrus | 18 | -88 | -8 | 18 | 24.3 | 6.8 |

^a^The F map was thresholded at F=5.3, p<0.001, uncorrected for multiple comparisons, with a cluster extent threshold k>8 contiguous voxels. BA=Brodmann areas. L = Left, R=Right hemisphere
