# Supplementary material for: Effect of Replacing Sugar with Non-Caloric Sweeteners in Beverages on the Reward Value after Repeated Exposure
Source: PLoS One. 2013 Nov 28;8(11):e81924. doi: 10.1371/journal.pone.0081924 (PMC3842969; doi:10.1371/journal.pone.0081924)
Supplement: Table S2 — Whole-brain statistical F-map with sweetener type and time as independent variables for tasting yoghurt drinks. (DOCX) [file pone.0081924.s003.docx]

**Table S2.** Whole-brain statistical F-map with sweetener type and time as independent variables for tasting yoghurt drinks ^a^

| **Activated region** | **Peak voxel coordinates** | | | **BA** | **F-score** | **z-score^a^** |
| --- | --- | --- | --- | --- | --- | --- |
|  | **x** | **y** | **z** |  |  |  |
| R Inferior frontal gyrus, orbital part | 45 | 38 | -14 | - | 6.2 | 3.5 |
| R Inferior frontal gyrus, triangular part | 60 | 26 | 1 | - | 9.3 | 4.4 |
| L Superior frontal gyrus | 21 | -1 | 58 | 6 | 6.1 | 3.5 |
| L Inferior frontal gyrus | -54 | 11 | 19 | 44 | 9.1 | 4.4 |
| L Middle frontal gyrus | -24 | 5 | 52 | 8 | 7.0 | 3.7 |
| L Parietal inferior gyrus | -54 | -28 | 37 | 2 | 12.7 | 5.2 |
| R Amygdala | 36 | 2 | -23 | 36 | 6.7 | 3.7 |
| Thalamus | 0 | -13 | 13 | - | 6.4 | 3.5 |
| L Middle Cingulum | -6 | -19 | 46 | - | 9.0 | 4.3 |
| R Precuneus | 21 | -55 | 52 | 7 | 6.8 | 3.7 |
| R Precentral gyrus | 60 | -16 | 37 | 43 | 10.1 | 4.6 |
| L Precentral gyrus | -48 | 5 | 40 | 6 | 6.8 | 3.7 |
| L Postcentral gyrus | -42 | -16 | 46 | 4 | 6.2 | 3.5 |
| R Rolandic Operculum | 63 | 2 | 10 | 48 | 12.0 | 5.1 |
| L Middle Temporal gyrus | -60 | -52 | 13 | 21 | 8.3 | 4.1 |
| R Middle Temporal gyrus | 45 | -73 | 10 | 37 | 6.1 | 3.4 |
| L Lingual gyrus | -18 | -88 | -11 | 18 | 13.2 | 5.3 |
| R Lingual gyrus | 24 | -88 | -8 | 18 | 16.7 | 5.9 |
| Cuneus | 9 | -85 | 19 | 18 | 15.9 | 5.8 |

^a^The F map was thresholded at F=5.2, p<0.001, uncorrected for multiple comparisons, with a cluster extent threshold k>8 contiguous voxels. BA=Brodmann areas. L = Left, R=Right hemisphere
