# Supplementary material for: Effect of Replacing Sugar with Non-Caloric Sweeteners in Beverages on the Reward Value after Repeated Exposure
Source: PLoS One. 2013 Nov 28;8(11):e81924. doi: 10.1371/journal.pone.0081924 (PMC3842969; doi:10.1371/journal.pone.0081924)
Supplement: Table S3 — Identified fROIs and results of analysis on mean beta value in each fROI. For tasting yoghurt drinks. (DOCX) [file pone.0081924.s004.docx]

**Table S3.** Identified fROIs and results of analysis on mean beta value in each fROI for tasting yoghurt drinks ^a^

|  | **fROI** | | **Peak voxel coordinates of fROI** | | | **BA** | **Cluster size (voxels)** | **F peak voxel** | **Main effect sweetener type** | **Sweetener type x**  **Time** |
| --- | --- | --- | --- | --- | --- | --- | --- | --- | --- | --- |
|  |  | | **x** | **y** | **z** |  |  |  | *p* | *p* |
|  | *OFC* | |  |  |  |  |  |  |  |  |
|  | | L Inferior Frontal gyrus | -42 | 38 | -14 | 47 | 25 | 4.4 | 0.13 | 0.44 |
|  | | R Inferior Frontal gyrus | 60 | 29 | -2 | - | 224 | 6.3 | 0.44 | 0.26 |
|  | | R Superior Temporal Pole | 30 | 11 | -23 | 38 | 17 | 3.9 | 0.95 | 0.64 |
|  | |  |  |  |  |  |  |  |  |  |
|  | *Amygdala* | |  |  |  |  |  |  |  |  |
|  | | L Amygdala | -27 | -1 | -23 | 36 | 36 | 5.1 | 0.67 | 0.22 |
|  | | R Amygdala | 27 | 2 | -23 | 34 | 38 | 6.7 | 0.49 | 0.34 |
|  | |  |  |  |  |  |  |  |  |  |
|  | *Thalamus* | |  |  |  |  |  |  |  |  |
|  | | Thalamus | 0 | -13 | 13 | - | 222 | 6.4 | 0.45 | 0.56 |
|  | |  |  |  |  |  |  |  |  |  |
|  | *Striatum* | |  |  |  |  |  |  |  |  |
|  | | L Caudate | -21 | 5 | 13 | - | 83 | 4.5 | 0.98 | 0.21 |
|  | | R Caudate | 12 | -4 | 13 | - | 14 | 3.7 | 0.89 | 0.22 |
|  | | L Putamen | -30 | -16 | 1 | 48 | 20 | 3.5 | 0.56 | 0.92 |
|  | | R Putamen | 36 | -16 | 1 | 48 | 16 | 3.4 | 0.31 | 0.44 |
|  | |  |  |  |  |  |  |  |  |  |
|  | *Cingulate Gyrus* | |  |  |  |  |  |  |  |  |
|  | | L Middle Cingulum | -6 | -16 | 43 | 23 | 214 | 8.2 | 0.17 | 0.29 |
|  | |  |  |  |  |  |  |  |  |  |
|  | *Hippocampus* | |  |  |  |  |  |  |  |  |
|  | | L Hippocampus | -33 | -13 | -23 | 20 | 19 | 5.6 | 0.21 | 0.56 |
|  | | R Hippocampus | 27 | -10 | -26 | 36 | 13 | 3.3 | 0.27 | 0.71 |
|  | |  |  |  |  |  |  |  |  |  |
|  | *Precentral Gyrus* | |  |  |  |  |  |  |  |  |
|  | | L Precentral gyrus | -60 | -19 | 37 | 43 | 455 | 8.4 | 0.44 | 0.36 |
|  | | L Precentral gyrus | -60 | 14 | 10 | 6 | 19 | 4.1 | 0.35 | **0.04** |
|  | | R Precentral gyrus | 63 | 2 | 10 | 48 | 492 | 12.0 | 0.56 | 0.17 |
|  | | R Precentral gyrus | 27 | -13 | 52 | 6 | 15 | 4.5 | 0.26 | 0.43 |
|  | | R Precentral gyrus | 21 | -28 | 58 | 6 | 38 | 4.2 | 0.70 | 0.64 |
|  | |  |  |  |  |  |  |  |  |  |
|  | *Taste map* | |  |  |  |  |  |  |  |  |
|  | | L Rolandic operculum | -51 | -19 | 16 | 48 | 39 | 5.7 | 0.48 | 0.24 |
|  | | R Rolandic operculum | 51 | -7 | 28 | 4 | 25 | 5.6 | 0.66 | 0.57 |

^a^The F map was thresholded at F=2.51, p<0.05, uncorrected for multiple comparisons, with a cluster extent threshold k>8 contiguous voxels. BA=Brodmann areas. L = Left, R=Right hemisphere
